# Complete genome sequence of the bile-resistant pigment-producing anaerobe *Alistipes finegoldii* type strain (AHN2437^T^)

**DOI:** 10.4056/sigs.3527032

**Published:** 2013-04-15

**Authors:** Konstantinos Mavromatis, Erko Stackebrandt, Christine Munk, Alla Lapidus, Matt Nolan, Susan Lucas, Nancy Hammon, Shweta Deshpande, Jan-Fang Cheng, Roxanne Tapia, Lynne A. Goodwin, Sam Pitluck, Konstantinos Liolios, Ioanna Pagani, Natalia Ivanova, Natalia Mikhailova, Marcel Huntemann, Amrita Pati, Amy Chen, Krishna Palaniappan, Miriam Land, Loren Hauser, Manfred Rohde, Sabine Gronow, Markus Göker, John C. Detter, James Bristow, Jonathan A. Eisen, Victor Markowitz, Philip Hugenholtz, Nikos C. Kyrpides, Hans-Peter Klenk, Tanja Woyke

**Affiliations:** 1 DOE Joint Genome Institute, Walnut Creek, California, USA; 2 Leibniz-Institute DSMZ - German Collection of Microorganisms and Cell Cultures, Braunschweig, Germany; 3 Los Alamos National Laboratory, Bioscience Division, Los Alamos, New Mexico, USA; 4 Biological Data Management and Technology Center, Lawrence Berkeley National Laboratory, Berkeley, California, USA; 5 Oak Ridge National Laboratory, Oak Ridge, Tennessee, USA; 6 HZI – Helmholtz Centre for Infection Research, Braunschweig, Germany; 7 University of California Davis Genome Center, Davis, California, USA; 8 Australian Centre for Ecogenomics, School of Chemistry and Molecular Biosciences, The University of Queensland, Brisbane, Australia

**Keywords:** Gram-negative, rod-shaped, non-sporulating, non-motile, mesophile, strictly anaerobic, chemoorganotrophic, *Rikenellaceae*, GEBA

## Abstract

*Alistipes finegoldii* Rautio *et al.* 2003 is one of five species of *Alistipes* with a validly published name: family *Rikenellaceae*, order *Bacteroidetes*, class *Bacteroidia*, phylum *Bacteroidetes*. This rod-shaped and strictly anaerobic organism has been isolated mostly from human tissues. Here we describe the features of the type strain of this species, together with the complete genome sequence, and annotation. *A. finegoldii* is the first member of the genus *Alistipes* for which the complete genome sequence of its type strain is now available. The 3,734,239 bp long single replicon genome with its 3,302 protein-coding and 68 RNA genes is part of the *** G****enomic* ***E****ncyclopedia of* ***Bacteria**** and* ***Archaea***** project.

## Introduction

Strain AHN2437^T^ (= DSM 17242 = CCUG 46020 = JCM 16770) is the type strain of *Alistipes finegoldii* [1,2]. This strain is one of several strains with similar properties [3] that were isolated mainly from pediatric patients with inflamed, gangrenous or non-inflamed appendices [4,5]. Though the type strain AHN2437^T^ resembled members of the *Bacteroides fragilis* group in bile-resistance and positive indole reaction, it was found, together with the type strain of *Bacteroides putredinis,* to form a separate phylogenetic lineage apart from authentic *Bacteroides* species [1]. The genus *Alistipes* was established to accommodate these two species and has subsequently been enlarged to encompass three additional species with validly published names and one with an effectively published name [6,7]. According to the position in ‘The All-Species Living Tree‘ 16S rRNA gene sequence dendrogram [8], the genus *Alistipes* is a sister clade of *Rikenella microfusus*, formerly *Bacteroides microfusus* [9,10], the two genera constituting the family *Rikenellaceae* [11,12]. Here we present a summary classification and a set of features for *A. finegoldii* AHN2437^T^ together with the description of the complete genomic sequencing and annotation.

## Classification and features

### 16S rDNA gene sequence analysis

A representative genomic 16S rRNA gene sequence of *A. finegoldii* AHN2437^T^ was compared using NCBI BLAST [13,14] under default settings (e.g., considering only the high-scoring segment pairs (HSPs) from the best 250 hits) with the most recent release of the Greengenes database [15] and the relative frequencies of taxa and keywords (reduced to their stem [16]) were determined, weighted by BLAST scores. The most frequently occurring genera were *Alistipes* (84.4%) and *Bacteroides* (15.6%) (19 hits in total). Regarding the three hits to sequences from members of the species, the average identity within HSPs was 98.7%, whereas the average coverage by HSPs was 98.0%. Regarding the nine hits to sequences from other members of the genus, the average identity within HSPs was 96.5%, whereas the average coverage by HSPs was 100.1%. Among all other species, the one yielding the highest score was *Alistipes shahii* (AB554233), which corresponded to an identity of 97.2% and an HSP coverage of 100.0%. (Note that the Greengenes database uses the INSDC (= EMBL/NCBI/DDBJ) annotation, which is not an authoritative source for nomenclature or classification.) The highest-scoring environmental sequence was AY643083 (Greengenes short name 'Isolation finegoldii blood two patients colon cancer *Alistipes finegoldii*; clone 3'), which showed an identity of 100.0% and an HSP coverage of 99.4%. The most frequently occurring keywords within the labels of all environmental samples which yielded hits were 'human' (11.5%), 'fecal' (8.1%), 'intestin' (5.5%), 'biopsi' (4.2%) and 'mucos' (4.0%) (231 hits in total). The most frequently occurring keywords within the labels of those environmental samples which yielded hits of a higher score than the highest scoring species were 'finegoldii' (18.2%), 'alistip, blood, cancer, colon, isol, patient, two' (9.1%) and 'fecal, human' (9.1%) (2 hits in total). These keywords are in accordance with the original isolation source of *A. finegoldii*.

**Figure 1:**
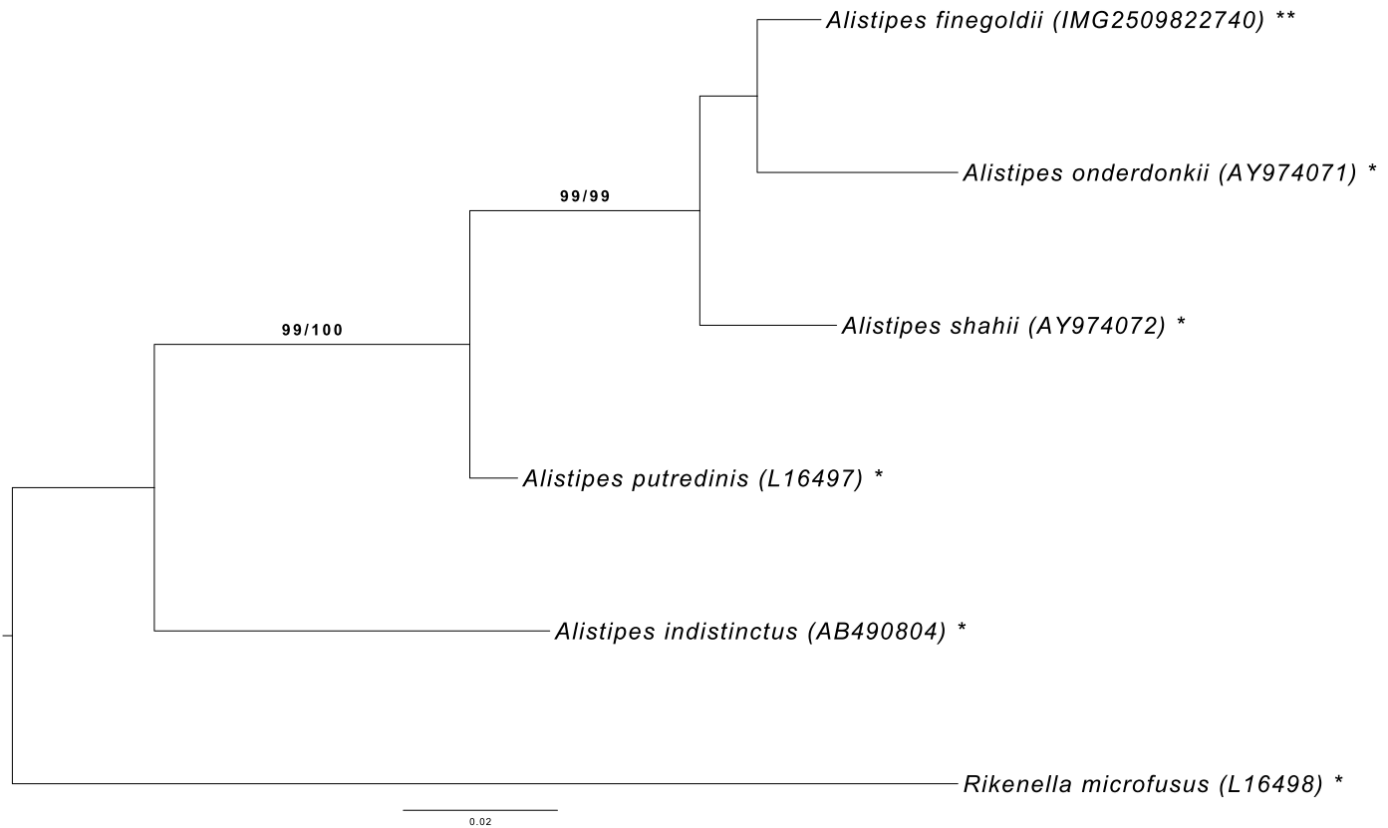
Phylogenetic tree highlighting the position of *A. finegoldii* relative to the type strains of the other species within the family *Rikenellaceae*. The tree was inferred from 1,432 aligned characters [17,18] of the 16S rRNA gene sequence under the maximum likelihood (ML) criterion [19]. Rooting was done initially using the midpoint method [20] and then checked for its agreement with the current classification (Table 1). The branches are scaled in terms of the expected number of substitutions per site. Numbers adjacent to the branches are support values from 1,000 ML bootstrap replicates [21] (left) and from 1,000 maximum-parsimony bootstrap replicates [22] (right) if larger than 60%. Lineages with type strain genome sequencing projects registered in GOLD [23] are labeled with one asterisk, those also listed as 'Complete and Published' with two asterisks. See also the species the not yet validly published names described together with their genome sequences in [6].

Figure 1 shows the phylogenetic neighborhood of *A. finegoldii* in a 16S rRNA gene based tree. The sequences of the two 16S rRNA gene copies in the genome differ from each other by ten nucleotides, and differ by up to ten nucleotides from the previously published 16S rRNA gene sequence (AY643083).

**Table 1:** Classification and general feature*s of A. finegoldii* AHN2437^T^ according to the MIGS recommendations [24].

MIGS ID	Property	Term	Evidence code
		Domain: *Bacteria*	TAS [25]
		Phylum *Bacteroidetes*	TAS [12,26]
		Class *Bacteroidia*	TAS [12,27]
	Current classification	Order *Bacteroidales*	TAS [12,28]
		Family *Rikenellaceae*	TAS [11,12]
		Genus *Alistipes*	TAS [1,2]
		Species *Alistipes finegoldii*	TAS [1,2]
MIGS-12	Reference for biomaterial	Rautio *et al*., 2003	TAS [1]
MIGS-7	Subspecific genetic lineage (strain)	AHN2437^T^	TAS [1]
	Gram stain	negative	TAS [1]
	Cell shape	rod-shaped	TAS [1]
	Motility	non-motile	TAS [1]
	Sporulation	non-sporulating	TAS [1]
	Temperature range	mesophile	TAS [1]
	Optimum temperature	37°C	TAS [1]
	Salinity	not reported	
MIGS-22	Relationship to oxygen	strictly anaerobe	TAS [1]
	Carbon source	not reported	
	Energy metabolism	chemoorganotroph	TAS [1]
MIGS-6	Habitat	probably human gut	TAS [1]
MIGS-6.2	pH	not reported	
MIGS-15	Biotic relationship	unknown	
MIGS-14	Known pathogenicity	none	TAS [1]
MIGS-16	Specific host	*Homo sapiens*	TAS [1]
MIGS-18	Health status of Host	unknown	
	Biosafety level	1	TAS [29]
MIGS-19	Trophic level	unknown	
MIGS-23.1	Isolation	human appendix tissue	TAS [1]
MIGS-4	Geographic location	Helsinki, Finland	TAS [1]
MIGS-5	Time of sample collection	1988	NAS
MIGS-4.1	Latitude	not reported	
MIGS-4.2	Longitude	not reported	
MIGS-4.3	Depth	not reported	
MIGS-4.4	Altitude	not reported	

Evidence codes: TAS: Traceable Author Statement (i.e., a direct report exists in the literature); NAS: Non-traceable Author Statement (i.e., not directly observed for the living, isolated sample, but based on a generally accepted property for the species, or anecdotal evidence). Evidence codes are from the Gene Ontology project [30].

### Morphology and physiology

**Figure 2:**
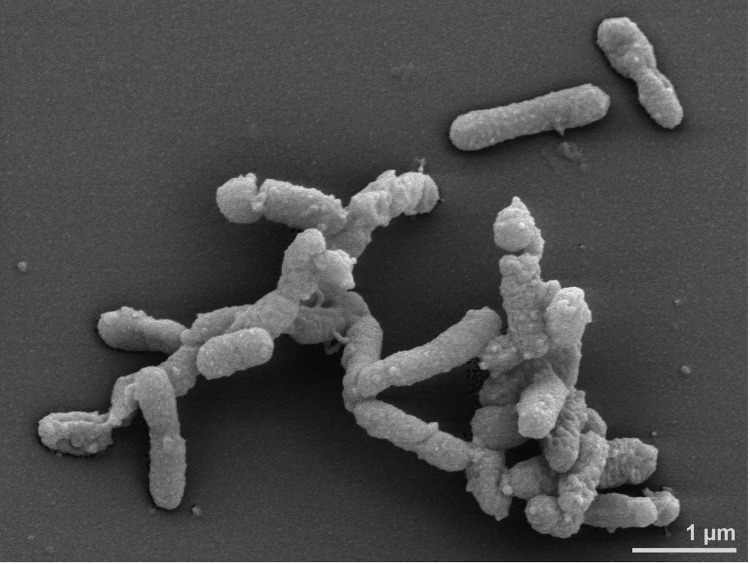
Scanning electron micrograph of *A. finegoldii* AHN2437^T^

Most members of *A. finegoldii* were isolated on *Bacteroides*-bile-esculin (BBE) agar, others on kanamycin/vancomycin laked blood agar. Cells stain Gram-negative, and are non-spore forming and rod-shaped with rounded ends (0.2 x 0.8 to 2 μm), mostly occurring singly, though longer filaments are observed occasionally (Figure 2). After 4 days growth on *Brucella* sheep blood agar colonies are 0.3–1.0 mm in diameter, circular, gray, translucent or opaque and weakly β-hemolytic. On laked rabbit blood agar colonies are light brown after 4 days incubation, turning reddish or chocolate brown after 10 days [1,3]. Growth temperature is 37°C [31]. The organism is strictly anaerobic, indole-positive, catalase-negative and grows in peptone-yeast extract-glucose containing 20% bile [1,3]. Nitrate is not reduced to nitrite, gelatin is liquefied and esculin hydrolysis is negative. Metabolism is fermentative, however, due to scanty growth on agar media and in liquid media, carbohydrate metabolism is difficult to evaluate. In PYG broth, succinic acid is the major end product, while acetic and propionic acids are minor products; isovaleric and lactic acids are sometimes produced in very small amounts. Acid- and alkaline phosphatases, N-acetyl-β-glucosaminidase, esterase, esterase lipase, α- and β-galactosidases, and α-glucosidase are detected in the API ZYM (bioMérieux) gallery, while no activity is detected for lipase C4, leucine/valine/cystine arylamidases, trypsin, β-glucuronidase, β-glucosidase or α-mannosidase. In addition, using Rosco diagnostic tablets (Rosco, Taastrup, Denmark), α-fucosidase is detected, but not β-xylosidase or trypsin. Strains are resistant to vancomycin (5 μg), kanamycin (1,000 μg), and colistin (10 μg). Susceptibility to penicillin varies and some strains produce β-lactamase (reaction for the type strain has not been specified) [1,3].

Strain AHN2437^T^ was isolated from a human appendiceal tissue sample. The habitat is not known but strains are probably members of the microflora of the human gut [1]. *A. finegoldii*-type organisms were identified by molecular methods as part of the microbiota of chicken guts [32] and they were detected in blood cultures from colon cancer patients [33].

### Chemotaxonomy

The major cellular fatty acid of strain AHN2437^T^ is *iso*-C_15:0_; smaller amounts (with 5 to 10% occurrence) are *anteiso*-C_15:0_, C_15:0_, C_16:0_, *iso*-C_17:0_, and one or both of C_17:0_ *iso*-3OH/C_18:2_ DMA. The mol% G+C of DNA is 57 [1,3]. No information is available for the peptidoglycan composition, isoprenoid composition, polar lipids or whole cell sugars.

## Genome sequencing and annotation

### Genome project history

This organism was selected for sequencing on the basis of its phylogenetic position [34], and is part of the *** G****enomic* ***E****ncyclopedia of* ***Bacteria**** and* ***Archaea***** project [35]. The genome project is deposited in the Genomes OnLine Database [23] and the complete genome sequence is deposited in GenBank. Sequencing, finishing and annotation were performed by the DOE Joint Genome Institute (JGI) using state of the art sequencing technology [46]. A summary of the project information is shown in Table 2.

**Table 2:** Genome sequencing project information

MIGS ID	Property	Term
MIGS-31	Finishing quality	Finished
MIGS-28	Libraries used	Three genomic libraries: one 454 pyrosequence standard library, one 454 PE library (11.0 kb insert size), one Illumina library
MIGS-29	Sequencing platforms	Illumina GAii, 454 GS FLX Titanium
MIGS-31.2	Sequencing coverage	133.3 × Illumina; 27.8 × pyrosequence
MIGS-30	Assemblers	Newbler version 2.3, Velvet version 1.0.13, Phrap version SPS - 4.24
MIGS-32	Gene calling method	Prodigal 1.4, GenePRIMP
	INSDC ID	CP003274
	GenBank Date of Release	June 8, 2012
	GOLD ID	Gc02257
	NCBI project ID	440775
	Database: IMG-GEBA	2509601035
MIGS-13	Source material identifier	DSM 17242
	Project relevance	Tree of Life, GEBA

### Growth conditions and DNA isolation

*A. finegoldii* strain AHN2437^T^, DSM 17242, was grown anaerobically in DSMZ medium 104 (PYG, supplemented with vitamin solution (see DSMZ medium 131)) [36] at 37°C. DNA was isolated from 1-1.5 g of cell paste using MasterPure Gram-positive DNA purification kit (Epicentre MGP04100) following the standard protocol as recommended by the manufacturer with modification st/LALM for cell lysis as described in Wu *et al*. 2009 [35]. DNA is available through the DNA Bank Network [37].

### Genome sequencing and assembly

The genome was sequenced using a combination of Illumina and 454 sequencing platforms. All general aspects of library construction and sequencing can be found at the JGI website [38]. Pyrosequencing reads were assembled using the Newbler assembler (Roche). The initial Newbler assembly consisting of 103 contigs in four scaffolds was converted into a phrap [39] assembly by making fake reads from the consensus, to collect the read pairs in the 454 paired end library. Illumina GAii sequencing data (500.5 Mb) was assembled with Velvet [40] and the consensus sequences were shredded into 2.0 kb overlapped fake reads and assembled together with the 454 data. The 454 draft assembly was based on 160.8 Mb 454 draft data and all of the 454 paired end data. Newbler parameters are -consed -a 50 -l 350 -g -m -ml 20. The Phred/Phrap/Consed software package [39] was used for sequence assembly and quality assessment in the subsequent finishing process. After the shotgun stage, reads were assembled with parallel phrap (High Performance Software, LLC). Possible mis-assemblies were corrected with gapResolution [38], Dupfinisher [41], or sequencing cloned bridging PCR fragments with subcloning. Gaps between contigs were closed by editing in Consed, by PCR and by Bubble PCR primer walks (J.-F. Chang, unpublished). A total of 696 additional reactions and 2 shatter libraries were necessary to close gaps and to raise the quality of the finished sequence. Illumina reads were also used to correct potential base errors and increase consensus quality using a software Polisher developed at JGI [42]. The error rate of the completed genome sequence is less than 1 in 100,000. Together, the combination of the Illumina and 454 sequencing platforms provided 161.1 × coverage of the genome. The final assembly contained 324,940 pyrosequence and 13,793,104 Illumina reads.

### Genome annotation

Genes were identified using Prodigal [43] as part of the DOE-JGI genome annotation pipeline [47], followed by a round of manual curation using the JGI GenePRIMP pipeline [44]. The predicted CDSs were translated and used to search the National Center for Biotechnology Information (NCBI) non-redundant database, UniProt, TIGR-Fam, Pfam, PRIAM, KEGG, COG, and InterPro databases. Additional gene prediction analysis and functional annotation was performed within the Integrated Microbial Genomes - Expert Review (IMG-ER) platform [45].

## Genome properties

**Figure 3:**
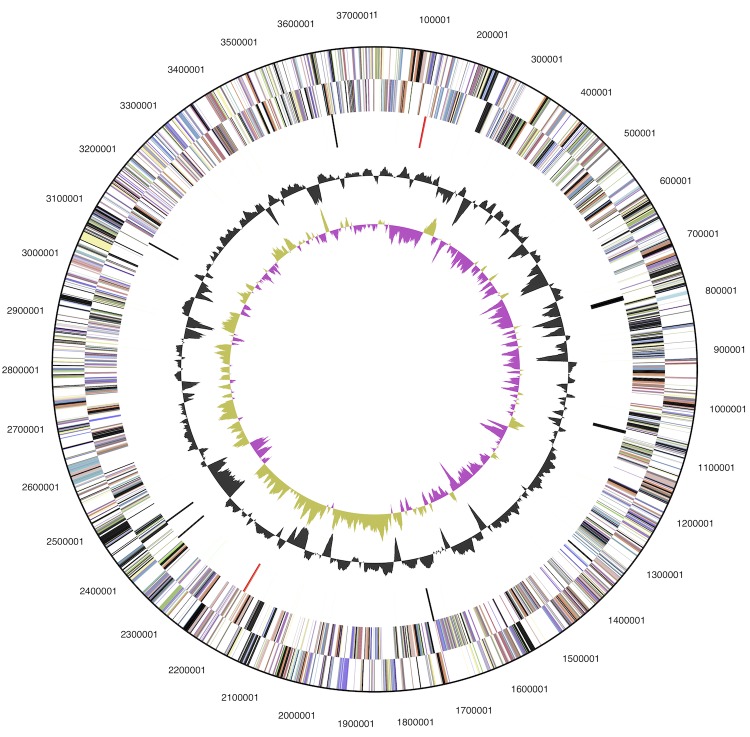
Graphical map of the chromosome. From outside to the center: Genes on forward strand (color by COG categories), Genes on reverse strand (color by COG categories), RNA genes (tRNAs green, rRNAs red, other RNAs black), GC content, GC skew (purple/olive).

The genome statistics are provided in Table 3 and Figure 3. The genome consists of one circular chromosome with a total length of 3,734,239 bp and a G+C content of 56.6%. Of the 3,302 genes predicted, 3,234 were protein-coding genes, and 68 RNAs; 121 pseudogenes were also identified. The majority of the protein-coding genes (62.0%) were assigned a putative function while the remaining ones were annotated as hypothetical proteins. The distribution of genes into COGs functional categories is presented in Table 4.

**Table 3:** Genome Statistics

Attribute	Value	% of Total
Genome size (bp)	3,734,239	100.00
DNA coding region (bp)	3,244,847	86.89
DNA G+C content (bp)	2,115,287	56.65
Number of replicons	1	
Extrachromosomal elements	0	
Total genes	3,302	100.00
RNA genes	68	2.06
rRNA operons	2	
tRNA genes	52	1.57
Protein-coding genes	3,234	97.94
Pseudo genes	121	3.66
Genes with function prediction	2,046	61.96
Genes in paralog clusters	1,627	49.27
Genes assigned to COGs	1,974	59.78
Genes assigned Pfam domains	2,183	66.11
Genes with signal peptides	967	29.29
Genes with transmembrane helices	642	19.44
CRISPR repeats	0	

**Table 4:** Number of genes associated with the general COG functional categories

**Code**	**Value**	**%age**	**Description**
J	144	6.8	Translation, ribosomal structure and biogenesis
A	...	...	RNA processing and modification
K	140	6.6	Transcription
L	214	10.0	Replication, recombination and repair
B	...	...	Chromatin structure and dynamics
D	36	1.7	Cell cycle control, cell division, chromosome partitioning
Y	...	...	Nuclear structure
V	40	1.9	Defense mechanisms
T	81	3.8	Signal transduction mechanisms
M	171	8.0	Cell wall/membrane biogenesis
N	7	0.3	Cell motility
Z	...	...	Cytoskeleton
W	...	...	Extracellular structures
U	56	2.6	Intracellular trafficking and secretion, and vesicular transport
O	77	3.6	Posttranslational modification, protein turnover, chaperones
C	127	6.0	Energy production and conversion
G	165	7.7	Carbohydrate transport and metabolism
E	141	6.6	Amino acid transport and metabolism
F	56	2.6	Nucleotide transport and metabolism
H	92	4.3	Coenzyme transport and metabolism
I	55	2.6	Lipid transport and metabolism
P	113	5.3	Inorganic ion transport and metabolism
Q	20	0.9	Secondary metabolites biosynthesis, transport and catabolism
R	259	12.2	General function prediction only
S	137	6.4	Function unknown
-	1,328	40.2	Not in COGs

## References

[1] Rautio M Eerola E Väisänen-Tunkelrott ML Molitoris D Lawson P Collins MD Jousimies-Somer H Reclassification of *Bacteroides putredinis* (Weinberg *et al*., 1937) in a new genus *Alistipes* gen. nov., as *Alistipes putredinis* comb. nov., and description of *Alistipes finegoldii* sp. nov., from human sources. Syst Appl Microbiol 2003; 26:182-188 10.1078/072320203322346029 12866844

[2] Validation List no. 94. Int J Syst Evol Microbiol 2003; 53:1701-1702 10.1099/ijs.0.03001-0 14657096

[3] Song Y Könönen E Rautio M Liu C Bryk A Eerola E Finegold SM *Alistipes onderdonkii* sp. nov. and *Alistipes shahii* sp. nov., of human origin. Int J Syst Evol Microbiol 2006; 56:1985-1990 10.1099/ijs.0.64318-0 16902041

[4] Rautio M Lönnroth M Saxén H Nikku R Väisänen ML Finegold SM Jousimies-Somer H Characteristics of an unusual anaerobic pigmented Gram-negative rod isolated from normal and inflamed appendices. Clin Infect Dis 1997; 25(Suppl 2):S107-S110 10.1086/516210 9310644

[5] Rautio M Saxén H Siitonen A Nikku R Jousimies-Somer H Bacteriology of histopathologically defined appedicitis in children. Pediatr Infect Dis 2000; 19:1078-1083 10.1097/00006454-200011000-00010 11099090

[6] Mishra AK Gimenez G Lagier JC Robert C Raoult D Fournier PE Genome sequence and description of *Alistipes senegalensis* sp. nov. Stand Genomic Sci 2012; 6:304-314 10.4056/sigs.2625821 23407265 PMC3558963

[7] Weinberg M, Nativelle R, Prévot AR. Les Microbes Anaérobies, Masson et Cie, Paris 1937; 1-1186.

[8] Munoz R Yarza P Ludwig W Euzéby J Amann R Schleifer KH Glöckner FO Rosselló-Móra R Release LTPs104 of the All-Species Living Tree. Syst Appl Microbiol 2011; 34:169-170 10.1016/j.syapm.2011.03.001 21497273

[9] Collins MD Shah HN Mitzuoka T Reclassification of *Bacteroides microfusus* (Kaneuchi and Mitsuoka) in a new genus *Rikenella*, as *Rikenella microfusus* comb. nov. Syst Appl Microbiol 1985; 6:79-81 10.1016/S0723-2020(85)80015-1

[10] Validation List N ° 18. Int J Syst Evol Microbiol 1985; 35:375-376

[11] Krieg NR, Staley JT, Brown DR, Hedlund BP, Paster BJ, Ward NL, Ludwig W, Whitman WB. Family III. *Rikenellaceae* fam. nov. *In:* Krieg NR, Staley JT, Brown DR, Hedlund BP, Paster BJ, Ward NL, Ludwig W, Whitman WB. (*eds*) Bergey’s Manual of Systematic Bacteriology, second edition, vol. 4 (The *Bacteroidetes, Spirochaetes, Tenericutes, Mollicutes), Acidobacteria, Fibrobacteres, Fusobacteria, Dictyoglomi, Gemmatimonadetes, Lentisphaerae, Verrucomicrobia, Chlamydiae*, and *Planctomycetes*), Springer, New York, 2011 p. 54.

[12] Validation List No 143. Int J Syst Evol Microbiol 2012; 62:1-4 10.1099/ijs.0.039487-0

[13] Altschul SF Gish W Miller W Myers EW Lipman DJ Basic local alignment search tool. J Mol Biol 1990; 215:403-410 2231712 10.1016/S0022-2836(05)80360-2

[14] Korf I, Yandell M, Bedell J. BLAST, O'Reilly, Sebastopol, 2003.

[15] DeSantis TZ Hugenholtz P Larsen N Rojas M Brodie EL Keller K Huber T Dalevi D Hu P Andersen GL Greengenes, a chimera-checked 16S rRNA gene database and workbench compatible with ARB. Appl Environ Microbiol 2006; 72:5069-5072 10.1128/AEM.03006-05 16820507 PMC1489311

[16] Porter MF. An algorithm for suffix stripping. *Program: electronic library and information systems* 1980; **14**:130-137.

[17] Lee C Grasso C Sharlow MF Multiple sequence alignment using partial order graphs. Bioinformatics 2002; 18:452-464 10.1093/bioinformatics/18.3.452 11934745

[18] Castresana J Selection of conserved blocks from multiple alignments for their use in phylogenetic analysis. Mol Biol Evol 2000; 17:540-552 10.1093/oxfordjournals.molbev.a026334 10742046

[19] Stamatakis A Hoover P Rougemont J A rapid bootstrap algorithm for the RAxML web-servers. Syst Biol 2008; 57:758-771 10.1080/10635150802429642 18853362

[20] Hess PN De Moraes Russo CA An empirical test of the midpoint rooting method. Biol J Linn Soc Lond 2007; 92:669-674 10.1111/j.1095-8312.2007.00864.x PMC7110036 32287391

[21] Pattengale ND Alipour M Bininda-Emonds ORP Moret BME Stamatakis A How many bootstrap replicates are necessary? Lect Notes Comput Sci 2009; 5541:184-200 10.1007/978-3-642-02008-7_13

[22] Swofford DL. PAUP*: Phylogenetic Analysis Using Parsimony (*and Other Methods), Version 4.0 b10. Sinauer Associates, Sunderland, 2002.

[23] Pagani I Liolios K Jansson J Chen IM Smirnova T Nosrat B Markowitz VM Kyrpides NC The Genomes OnLine Database (GOLD) v.4: status of genomic and metagenomic projects and their associated metadata. Nucleic Acids Res 2012; 40:D571-D579 10.1093/nar/gkr1100 22135293 PMC3245063

[24] Field D Garrity G Gray T Morrison N Selengut J Sterk P Tatusova T Thomson N Allen MJ Angiuoli SV The minimum information about a genome sequence (MIGS) specification. Nat Biotechnol 2008; 26:541-547 10.1038/nbt1360 18464787 PMC2409278

[25] Woese CR Kandler O Wheelis ML Towards a natural system of organisms: proposal for the domains *Archaea, Bacteria*, and *Eucarya.* Proc Natl Acad Sci USA 1990; 87:4576-4579 10.1073/pnas.87.12.4576 2112744 PMC54159

[26] Krieg NR, Ludwig W, Euzéby J, Whitman WB. Phylum XIV. *Bacteroidetes* phyl. nov. *In:* Krieg NR, Staley JT, Brown DR, Hedlund BP, Paster BJ, Ward NL, Ludwig W, Whitman WB. (*eds*) Bergey’s Manual of Systematic Bacteriology, second edition, vol. 4 (The *Bacteroidetes, Spirochaetes, Tenericutes, Mollicutes), Acidobacteria, Fibrobacteres, Fusobacteria, Dictyoglomi, Gemmatimonadetes, Lentisphaerae, Verrucomicrobia, Chlamydiae*, and *Planctomycetes*), Springer, New York, 2011 p. 25.

[27] Krieg NR. Class I. *Bacteroidia* class. nov. *In:* Krieg NR, Staley JT, Brown DR, Hedlund BP, Paster BJ, Ward NL, Ludwig W, Whitman WB. (*eds*) Bergey’s Manual of Systematic Bacteriology, second edition, vol. 4 (The *Bacteroidetes, Spirochaetes, Tenericutes, Mollicutes), Acidobacteria, Fibrobacteres, Fusobacteria, Dictyoglomi, Gemmatimonadetes, Lentisphaerae, Verrucomicrobia, Chlamydiae*, and *Planctomycetes*), Springer, New York, 2011 p. 25.

[28] Krieg NR. Order I. *Bacteroidales* ord. nov. *In:* Krieg NR, Staley JT, Brown DR, Hedlund BP, Paster BJ, Ward NL, Ludwig W, Whitman WB. (*eds*) Bergey’s Manual of Systematic Bacteriology, second edition, vol. 4 (The *Bacteroidetes, Spirochaetes, Tenericutes, Mollicutes), Acidobacteria, Fibrobacteres, Fusobacteria, Dictyoglomi, Gemmatimonadetes, Lentisphaerae, Verrucomicrobia, Chlamydiae*, and *Planctomycetes*), Springer, New York, 2011 p. 25.

[29] BAuA. 2010, Classification of *Bacteria* and *Archaea* in risk groups. http://www.baua.de TRBA 466, p. 19.

[30] Ashburner M Ball CA Blake JA Botstein D Butler H Cherry JM Davis AP Dolinski K Dwight SS Eppig JT Gene ontology: tool for the unification of biology. Nat Genet 2000; 25:25-29 10.1038/75556 10802651 PMC3037419

[31] DSMZ catalogue of strains, Braunschweig, Germany. (http://www.dsmz.de/catalogues/catalogue-microorganisms.html)

[32] Torok VA Hughes RJ Mikkelsen LL Perez-Maldonado R Balding K MacAlpine R Percy NJ Ophel-Keller K Identification and characterization of potential performance-related gut microbiotas in broiler chickens across various feeding trials. Appl Environ Microbiol 2011; 77:5868-5878 10.1128/AEM.00165-11 21742925 PMC3165380

[33] Fenner L Roux V Ananian P Raoult D *Alistipes finegoldii* in blood cultures from colon cancer patients. Emerg Infect Dis 2007; 13:1260-1262 10.3201/eid1308.060662 17953110 PMC2828066

[34] Klenk HP Göker M *En route* to a genome-based classification of *Archaea* and *Bacteria?* Syst Appl Microbiol 2010; 33:175-182 10.1016/j.syapm.2010.03.003 20409658

[35] Wu D Hugenholtz P Mavromatis K Pukall R Dalin E Ivanova NN Kunin V Goodwin L Wu M Tindall BJ A phylogeny-driven genomic encyclopedia of *Bacteria* and *Archaea.* Nature 2009; 462:1056-1060 10.1038/nature08656 20033048 PMC3073058

[36] List of growth media used at DSMZ: http://www.dsmz.de/catalogues/catalogue-microorganisms/culture-technology/list-of-media-for-microorganisms.html

[37] Gemeinholzer B Dröge G Zetzsche H Haszprunar G Klenk HP Güntsch A Berendsohn WG Wägele JW The DNA Bank Network: the start from a German initiative. Biopreserv Biobank 2011; 9:51-55 10.1089/bio.2010.0029 24850206

[38] JGI website. http://www.jgi.doe.gov/

[39] The Phred/Phrap/Consed software package. http://www.phrap.com

[40] Zerbino DR Birney E Velvet: algorithms for de novo short read assembly using de Bruijn graphs. Genome Res 2008; 18:821-829 10.1101/gr.074492.107 18349386 PMC2336801

[41] Han C, Chain P. Finishing repeat regions automatically with Dupfinisher. *In:* Proceeding of the 2006 international conference on bioinformatics & computational biology. Arabnia HR, Valafar H (*eds*), CSREA Press. June 26-29, 2006:141-146.

[42] Lapidus A, LaButti K, Foster B, Lowry S, Trong S, Goltsman E. POLISHER: An effective tool for using ultra short reads in microbial genome assembly and finishing. AGBT, Marco Island, FL, 2008.

[43] Hyatt D Chen GL LoCascio PF Land ML Larimer FW Hauser LJ Prodigal: prokaryotic gene recognition and translation initiation site identification. BMC Bioinformatics 2010; 11:119 10.1186/1471-2105-11-119 20211023 PMC2848648

[44] Pati A Ivanova NN Mikhailova N Ovchinnikova G Hooper SD Lykidis A Kyrpides NC GenePRIMP: a gene prediction improvement pipeline for prokaryotic genomes. Nat Methods 2010; 7:455-457 10.1038/nmeth.1457 20436475

[45] Markowitz VM Ivanova NN Chen IMA Chu K Kyrpides NC IMG ER: a system for microbial genome annotation expert review and curation. Bioinformatics 2009; 25:2271-2278 10.1093/bioinformatics/btp393 19561336

[46] Mavromatis K Land ML Brettin TS Quest DJ Copeland A Clum A Goodwin L Woyke T Lapidus A Klenk HP The fast changing landscape of sequencing technologies and their impact on microbial genome assemblies and annotation. PLoS ONE 2012; 7:e48837; . 10.1371/journal.pone.0048837 23251337 PMC3520994

[47] Mavromatis K Ivanova NN Chen IM Szeto E Markowitz VM Kyrpides NC The DOE-JGI Standard operating procedure for the annotations of microbial genomes. Stand Genomic Sci 2009; 1:63-67 10.4056/sigs.632 21304638 PMC3035208

